# The Prevalence of Chronic Mouth Infections and Their Management*Read before the Section on Stomatology at the Sixty-Seventh Annual Session of the American Medical Association, Detroit, June, 1916. Printed in *Journal A. M. A.*, Sept. 16, 1916.

**Published:** 1916-10

**Authors:** Frederick B. Moorehead

**Affiliations:** Chicago


### THE PREVALENCE OF CHRONIC MOUTH INFECTIONS AND THEIR MANAGEMENT.*

BY FREDERICK B. MOOREHEAD, M.D., D.D.S., CHICAGO.

An examination of the histories of 498 cases of chronic arthritis in the service of Dr. Frank Billings at the Presbyterian Hospital, Chicago, furnishes valuable information on the incidence of chronic mouth infections. Of this number 332 had careful Roentgen-ray mouth examinations; 1,132 periapical infections ranging from small areas of rarefaction to gross bone lesions were recorded; 994 of the teeth involved had been treated, with varying degrees of root-canal fillings, and 138 teeth showed no evidence of root-canal treatment. The percentage of patients in this group showing definite Roentgen-ray evidence of chronic alveolar abscess was eighty-nine; 1,342 teeth were involved in varying degrees of pyorrhea, the diagnosis being based largely on the Roentgen-ray evidence of peridental membrane destruction. Seventy-six per cent. of the patients showed well defined pyorrhea, many of them well advanced with marked suppuration.

A second group of patients from Dr. Billings' service, seventy in number, excluding all arthritics, showed chronic

* Read before the Section on Stomatology at the Sixty-Seventh Annual Session of the American Medical Association, Detroit, June, 1916. Printed in *Journal A. M. A.*, Sept. 16, 1916.

alveolar abscess in seventy-four per cent. The list included myositis, neuritis, goiter, asthma, nephritis, etc. Forty-nine per cent. had pyorrhea. A third group taken from 150 private office records showed chronic alveolar abscess in sixty-nine per cent.

In determining the incidence of chronic alveolar abscess in patients suffering from chronic disease, one should limit his investigations to adults. Other foci of infection are more likely to be responsible for chronic disease in young people in whom the tonsil undoubtedly is more often responsible than the teeth.

While pulpless teeth are met with in young people, the percentage is quite low compared to those of middle life. This is more largely true of pyorrhea than chronic alveolar abscess. Pyorrhea before twenty-five years of age is comparatively rare. The danger both from chronic alveolar abscess and pyorrhea decreases with advancing age, and the loss of teeth. One may regard, therefore, the period from twenty-five to sixty as the period of greatest danger.

An edentulous jaw or partially edentulous jaw, where the remaining teeth are free from infection, does not absolve the mouth from responsibility. The removal of part or all of the teeth involved in a chronic infection may serve to clean up the mouth of a patient whose tissues elsewhere have suffered permanent harm, and without effecting a cure. The fact that a mouth at the time of examination may be free from infection does not exclude such a mouth from responsibility any more than the removal of infected teeth insures the repair of permanently damaged joints or other structures.

Such an argument, however, does not absolve one from the responsibility of removing infection whenever it is discovered, whether in recent or long standing cases. The 718 cases cited illustrate the incidence of chronic mouth infection in three groups of cases. The first group of 498 cases were all chronic arthritics. The second group of seventy were chronic infections, none of them suffering from joint lesions. The third group of 150 were office cases, referred for mouth examination because of some systemic disease.

In the first group eighty-nine per cent. had alveolar abscess; the second group seventy-four per cent., the third group sixty-nine per cent.

The appearance of eighty-nine per cent. of chronic alveolar abscess in so large a group of hospital patients is probably more than presumptive clinical evidence, particularly when viewed in the light of results following treatment and animal experimentation.

In the light of our present knowledge these facts must constitute an eloquent predication. An interesting feature of our study of the frequency of chronic mouth infections was the discovery that the poorer classes had relatively fewer chronic abscesses than the middle and well-to-do classes. The reason is not difficult to find—the poor people, including foreigners, usually have aching teeth treated by early extraction.

Considering the manner of infection, chronic alveolar abscess may be classified as primary and secondary; primary, those in which infection occurs through the root canal from an infected pulp; from faulty technic in root canal treatment; from a failure to adequately seal the root canal and pulp chamber in the introduction of permanent filling materials. Those infections classed as secondary are blood borne, the predisposing cause being a lowered resistance in the periapical tissue brought about, first, by the careless use of arsenic as a devitalizing agent, and, secondly, by the indiscriminate use of irritating agents in the treatment of root canals. The irritation set up in the tissues at the apex of the root results in a stimulation and multiplication of the fixed tissue cells, and the end result is scar tissue, which interferes with normal circulation. The resistance of such areas is relatively low. A periapical infection in a vital tooth can scarcely occur. These secondary infections may be largely represented by the principle of asepsis rather than antisepsis. More good and less harm is accomplished by surgical cleanliness than by the use of antiseptics and disinfectants.

The relative danger of chronic abscess compared to pyorrhea is difficult to measure. While a closed suppurating cavity is more serious than an open one, the quantity of infection is apt to be very much greater in pyorrhea.

Pyorrhea may be looked on as the lesser of two evils, but not to any degree of tolerance.

TREATMENT.

The object of treatment is twofold: first, to save the teeth, and second, to rid the mouth of infection. Whatever form of treatment may be undertaken, nothing short of restoring the tissues to a state of health can be considered satisfactory.

A discussion of the various methods employed in the treatment of pyorrhea would for the present purpose be without profit. No fault can be found with an honest, intelligent effort to save teeth involved in a chronic suppurative pericementitis.

In carefully selected cases in the mouths of patients who are in a state of good health, one may properly undertake the treatment of teeth which are not seriously damaged. Where a large portion of the peridental membrane has been destroyed, no permanent cure need be expected. The process of repair in this class consists in the formation of connective tissue—scar tissue—in which the circulation of blood is very limited and which does not form a biologic union between the cementum and alveolar process. No permanency need be expected of repair of this type.

The fibers of the peridental membrane are attached to the cementum by the cementoblasts. Destroyed by a chronic suppurative pericementitis, the cementoblasts probably do not regenerate to any appreciable extent. The cementoblasts being destroyed, there is no hope of union between the cementum and surrounding tissues, except by scar tissue. There is no continuity of tissue in this type of repair, and reinfection is likely to follow.

Well selected cases in the hands of specially trained men undoubtedly yield gratifying results; but the number of men thus qualified form a small percentage of the dental profession, and a great many people are afflicted with chronic suppurative pericementitis.

Recently emetin has been rather extensively advocated and used in the treatment of this condition on the assumption that the endameba was the essential cause. This has been based upon a rather far fetched analogy. No one to our knowledge has demonstrated by animal experiments, or even convincing clinical evidence, that the endameba bears anything more than perhaps a symbiotic relationship to the infection. While we can not at this time enter into a detailed discussion of the subject, we can not refrain from expressing the opinion that the use of emetin as an experiment has been a failure.

The treatment of chronic alveolar abscess falls naturally into two groups: (1) conservative, and (2) radical.

The first type saves the tooth, and, theoretically at least, removes the infection. The second type removes the tooth and is the only method which removes infection in all cases. In the first type there is involved the opening of each root canal to its apical foramen, and complete disinfection and filling with a neutral substance which shall seal the canal at the foramen, and shall not be affected by moisture or cause irritation to the surrounding tissue. Theoretically these are the requirements. Practically they are realized in a minor degree.

Allowing for natural conditions and body defenses, probably not more than twenty-five per cent. of all root canals treated may be considered safe. This statement embraces general practice and does not particularize the few teeth treated by a few specialists.

At present there are not enough dentists to care for the people, and while the poor and ignorant have been automatically excluded in the past, they must now be dealt with, because of our new knowledge of chronic infection, on the same basis as the better classes.

Perhaps not over ten per cent. of dentists may be considered competent to undertake difficult root canal operations. Surely not over ten per cent. of the people can afford to pay for the services of a dentist who has acquired high skill in root canal technic. If, then, there are too few dentists to take care of all the people, and all the people must be cared for in the matter of infections, and only ten per cent. of the dentists may be called on to save teeth involved in chronic infections, and only ten per cent. can afford the services of a specialist, after discounting the successes of specialists in root canal technic, one is confronted with the practical fact that most of the people are seriously involved.

One may postulate that only certain chronic infections are dangerous; but who shall discriminate and say, "This chronic abscess or pyorrhea is harmless and this one is harmful?" When in doubt and the patient is ill, which shall be sacrificed, the tooth, or a possible opportunity for the patient's recovery? No doubt teeth are being extracted which do not bear a causal relationship to general infection; but it certainly is the lesser of two evils to remove the tooth. In so doing one is at least performing a reasonable service in an attempt to bring back health, and whether a patient is well or ill it is never justifiable to allow any infection to remain anywhere in the body which may be removed with as little loss or discomfort as the extraction of a tooth. The functional value of an infected tooth can not compensate for the harm it may cause.

Every tooth should be saved which may be made to serve the patient better than an artificial substitute, provided we interpret the quality of service in the light of the health and comfort of the patient. Conservative dentistry is strongly urged when its objective is general physical well being. Saving teeth which perpetuate chronic infection is not conservative dentistry, but destructive dentistry. The chief object of dentistry must always be the maintenance of health. The teeth must not be looked on as independent structures but as interdependent structures. Viewed as tissues intimately associated with health and disease, the teeth and their adnexa can not be dealt with in the abstract. One may undertake conservative measures in the mouths of patients who are in good health and whose general body defenses are good. Such cases, however, must be checked up and not dismissed until the teeth in question are either free from infection, or, in case of failure of treatment, have been removed. While at a given time a patient may be in a state of health, one may not presume on good health, and permit conditions to exist which he would not permit in the mouths of those in a state of ill health.

Because of general serious conditions one feels obliged to extract teeth which might be saved if the health of the patient would justify the necessary time for treatment. Teeth may always be extracted, and every reasonable effort should be made to save them when local conditions indicate that they may be saved, and the patient's interests are in no way jeopardized by the process.

While the Roentgen ray is relative, not absolute, we are dependent on it for our estimate of tissue loss both in chronic suppurative pericementitis and chronic abscess. The interpretation of Roentgen-ray films is a fine art, but no amount of experience will save one from occasional surprises and disappointments. A large granuloma, for example, may involve only the spongiosum, and where the external and internal bony plates are thick and dense, the Roentgen ray will not define the area.

To differentiate between an active infection and a rarefied area which has been disinfected and is undergoing repair is difficult or even impossible, especially in a dense field.

The relation of tooth roots to rarefied areas may quite obscure a lesion where the exposure has been made from only one angle. Even though unsatisfactory at times, the Roentgen ray is an indispensable factor in the diagnosis of bone lesions associated with the teeth, and the percentage of failures or mistakes in diagnosis is relatively small.

The very fact that clinical experience demonstrates occasional positive and negative errors only serves to make one careful not to overlook the danger of doubtful cases. The apparent size of a diseased area bears no relation whatever to its possible danger. Chronic infections are dangerous because of quality, not quantity. When conservative treatment is undertaken, the Roentgen ray should be used, first to demonstrate successful root-canal fillings, and second, to check up on the process of repair. Partially healed foci must be looked on with suspicion. Residual infection, though limited in extent, following either therapeutic or surgical measures, may be quite as dangerous as before treatment. No dependence whatever may be placed on subjective symptoms. Through active therapeutic measures a chronic alveolar abscess with a discharging sinus may quiet down, the sinus close, and the overlying tissue become normal. From clinical evidence alone such cases have been classed as cured. In the absence of a roentgenogram no definition of repair can be made. Frequently these cases do not heal, and are much more serious than before treatment, the treatment rarely serving to convert an open infection into a closed one.

SUMMARY.

1. The incidence of chronic mouth lesions in a group of over 700 carefully analyzed cases, showing percentages ranging from sixty-nine to eighty-nine per cent., must be looked on as more or less serious evidence.

2. The overwhelming majority of chronic abscesses being associated with previously treated root canals serves to emphasize the importance of root-canal technic.

3. Both in diagnosis and in determining the extent of tissues lost, the Roentgen ray is paramount.

4. The involvement of the peridental membrane is the crux in deciding between conservative and radical treatment.

5. Faulty root-canal technic, the careless use of arsenic as devitalizing agent, and irritating drugs in the treatment of root canals are strong predisposing factors of chronic alveolar abscess.

6. In carefully selected cases, conservative measures should be employed both in the treatment of chronic abscess and chronic suppurative pericementitis.

7. Where root canals have been disinfected and filled, portions of roots resected, etc., the process of repair should be checked up by roentgenograms made at frequent intervals.

8. Regardless of whatever form of treatment may be employed, the removal of infection is imperative in all cases, whether the patient at the time may be well or ill.

Where the health, comfort and usefulness of a patient are to be weighed over against a tooth, or even all the teeth, the greater interests of the patient must be preserved.

